# Evidence for exposure to and infection with H5 clade 2.3.4.4b highly pathogenic avian influenza viruses among polar bears inhabiting Alaska

**DOI:** 10.1016/j.isci.2026.117736

**Published:** 2026-09-22

**Authors:** Andrew M. Ramey, Lindsey S. Mangipane, Todd C. Atwood, Raphaela Stimmelmayr, David S. Rotstein, Christina A. Ahlstrom, Kristin Simac, Sarah M. Coburn, Mia Kim Torchetti, David E. Stallknecht, Rebecca L. Poulson

**Affiliations:** 1 U.S. Geological Survey, Alaska Science Center, Anchorage, AK, USA; 2 U.S. Fish and Wildlife Service, Marine Mammals Management, Anchorage, AK, USA; 3 North Slope Borough, Department of Wildlife Management, Utqiağvik, AK, USA; 4 Institute of Arctic Biology, University of Alaska Fairbanks, Fairbanks, AK, USA; 5 Marine Mammal Pathology Services, Olney, MD, USA; 6 Alaska Department of Environmental Conservation, Anchorage, AK, USA; 7 U.S. Department of Agriculture, National Veterinary Services Laboratories, Ames, IA, USA; 8 Southeastern Cooperative Wildlife Disease Study, University of Georgia, Athens, GA, USA

**Keywords:** arctic, bird flu, disease, pathology, serology, North America, *Ursus maritimus*, wildlife

## Abstract

Highly pathogenic avian influenza viruses (HPAIVs) have become widespread among wildlife including within the Arctic. We summarize histopathological findings for three cases of HPAIV infection among polar bears (*Ursus maritimus*) inhabiting Alaska, genomically characterize viruses causing disease, and assess probable non-lethal exposures. Polar bears found dead and infected with HPAIVs exhibited meningoencephalomyelitis and neuronal necrosis, consistent with neurologic disease. Genomic sequence information obtained for H5N1 and H5N5 subtype HPAIVs associated with two cases supported epidemiologically independent spillovers. Serologic testing provided evidence for antibodies reactive to H5 and N1 antigens among most polar bears inhabiting Alaska during 2023–2024, consistent with contemporary non-lethal exposures, whereas antibodies were not found among bears sampled during 2013. These data indicate that HPAIV infection may represent an emerging threat to polar bear health within the Alaska Arctic; though, population immunity seems to be building and may protect this species from widespread demographic impacts of disease.

## Highlights

• Three dead polar bears were infected with highly pathogenic avian influenza viruses
• Pathology indicated neurologic disease
• Genomic sequence data supported epidemiologically independent viral spillovers
• Serology indicated non-lethal exposures among apparently healthy animals

## Graphical abstract

**Figure:**
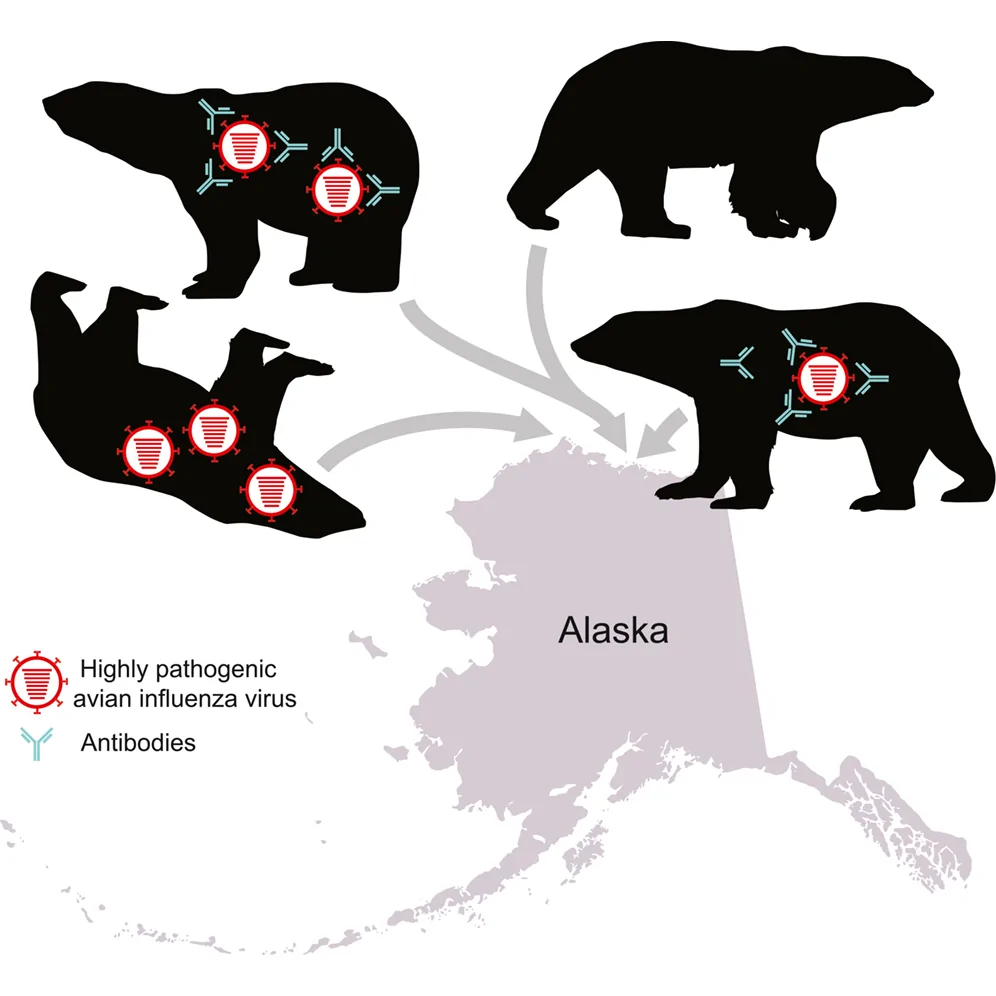

## Teaser

wildlife microbiology; wildlife pathology; virology

## Introduction

Since emergence circa 1996, highly pathogenic avian influenza viruses (HPAIVs) of the goose/Guangdong genetic lineage have evolved from geographically restricted pathogens affecting domestic poultry within East Asia to globally widespread and genomically diverse viruses causing disease and mortality among wild birds, marine and terrestrial mammals, peridomestic animals, poultry, livestock, and less commonly, humans.^1^^,^^2^^,^^3^^,^^4^^,^^5^^,^^6^^,^^7^^,^^8^ Geographic expansion of these HPAIVs has been particularly pronounced since 2021 as viruses of H5 clade 2.3.4.4b (2.3.4.4b) spread from Africa and Eurasia to North America, South America, Antarctica, and Australia, diversifying via antigenic shift and drift en route.^2^^,^^9^^,^^10^ During this most recent period of geographic and host species expansion, 2.3.4.4b HPAIVs have predominately been reported to be of the H5N1 combined subtype, though other reassortant viral subtype combinations (e.g., H5N5) have been sporadically detected.^8^ Relatively few ecosystems on earth remain unaffected by 2.3.4.4b HPAIVs as exemplified by recent detections among free-ranging wildlife in some of the most remote regions of the planet.^11^^,^^12^

One of the more prominent recent detections of HPAIVs among wildlife from a remote region was the confirmation of 2.3.4.4b HPAIV from a deceased polar bear (*Ursus maritimus*) cub found dead on the Arctic coast of Alaska (United States of America; USA) near the community of Utqiağvik in 2023.^13^ Polar bears are largely ice-dependent apex predators that rely upon seals for the majority of their dietary needs.^14^^,^^15^ However, polar bears in Alaska and elsewhere are increasingly using land during periods of seasonal sea ice retreat^16^^,^^17^ where they may consume birds and eggs, scavenge on marine mammal carcasses, feed on a variety of plants, or utilize other available food sources.^14^ Polar bears that spend time on land have previously been associated with increased exposures to pathogens which could be related to dietary exposures, terrestrial denning, or other factors.^18^^,^^19^^,^^20^ However, varying trends in pathogen exposure among sampling locations indicates that local ecological context is likely important.^18^^,^^19^^,^^20^ The route through which the polar bear cub found dead was exposed to and infected with 2.3.4.4b HPAIV remains unclear.^13^

Aside from the single published detection of 2.3.4.4b HPAIV from a polar bear in 2023,^13^ there are limited historical data to indicate that exposure of this species to avian origin influenza viruses is common. A prior serosurvey found no evidence for antibodies reactive to the influenza virus nucleoprotein among 100 polar bears captured along the Beaufort Sea coast of Alaska during 2013–2016.^21^ Another investigation identified a single individual with antibodies reactive to the influenza virus nucleoprotein among 26 polar bears captured at Franz Josef Land and Novaya Zemlya, Russia during 2010–2011.^22^ A polar bear from a zoologic collection in Germany was also identified to have antibodies reactive to the influenza virus nucleoprotein following death in 2011.^23^

Since the initial detection of a polar bear infected with 2.3.4.4b HPAIV during 2023, two additional cases of deceased polar bears associated with HPAIVs have been identified in Alaska (i.e., during 2024 and 2025), though formal descriptions of diagnostic findings have not previously been summarized in the published literature, nor have the causative agents of disease been genomically characterized. Furthermore, there is an apparent lack of publicly available contemporary information on non-lethal exposures of polar bears inhabiting Alaska to 2.3.4.4b HPAIVs. These data gaps preclude rigorous inference on exposure pathways, case fatality rates, and implications to polar bear population health. In this study, we aimed to bridge these data gaps by formally describing the 2024 and 2025 cases of polar bears affected by HPAIVs along Alaska’s Southern Beaufort Sea, providing information on the genomic context for the viruses causing disease, and assessing probable non-lethal exposures of polar bears to HPAIVs within the greater region.

## Results

### Results summary

Through postmortem examination, as well as histopathological and immunohistochemical analysis, three polar bears found dead and infected with 2.3.4.4b HPAIVs were identified as exhibiting signs of acute, inflammatory, and destructive processes in the central nervous system. Genomic sequence information was obtained for H5N1 and H5N5 subtype HPAIVs associated with two of these fatal cases, neither of which contained molecular markers reported to be indicative of mammalian adaptation, altered virulence, or strong drug resistance. Serologic testing provided evidence for antibodies reactive to 2.3.4.4b H5 and N1 antigens among most polar bears inhabiting coastal and nearshore habitats of Alaska during 2023–2024, whereas antibodies reactive to these same antigens were not found among bears sampled during 2013.

### Confirmed infections with HPAIV among deceased polar bears

The first case of a polar bear ultimately confirmed to be affected by 2.3.4.4b HPAIV, was a cub of the year (i.e., <1 year old) found dead near Point Barrow, Alaska (71.3836 °N, 156.4669 °W) on 27 August 2023 (Figure 1). This case was the subject of a previous report,^13^ therefore, detailed description is not provided here. However, histopathologic findings are summarized (Table 1) for comparative purposes.

**Figure 1:**
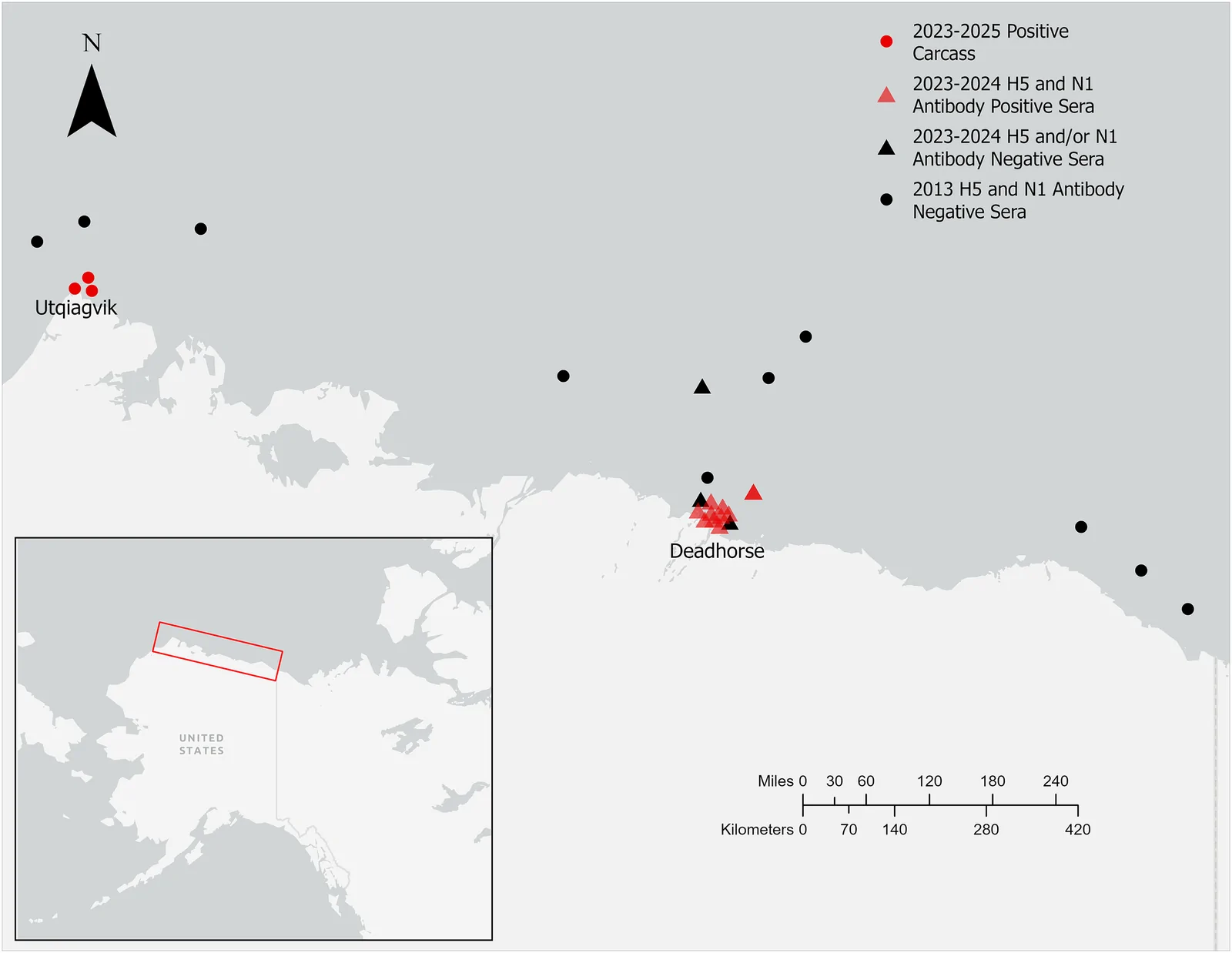
Map depicting Alaska (inset) and locations along the Arctic Beaufort Sea coast where samples were collected from polar bears that were later analyzed and summarized for this investigation Black icons represent locations at which sera samples were collected in 2013 (circles) or in 2023–2024 (triangles) which were inferred to be H5 and/or N1 antibody negative. Red icons represent locations at which tissue, swab, or sera samples were collected during 2023–2025 from which HPAIVs (circles) or H5 and N1 antibodies (triangles) were identified. Overlapping shapes were jittered to facilitate visualization. The relative locations of Utqiağvik and Deadhorse are provided for reference. Figure was generated using Esri light gray base map (US Edition) 20 February 2025; https://www.arcgis.com/sharing/rest/content/items/12124841a631489fb71aa1c0c8952c49/resources/styles/root.json.

**Table 1:** Histopathologic findings from three positive polars confirmed to be affected by H5 clade 2.3.4.4b HPAIV in Alaska, 2023–2025

System	Histopathologic finding	Number with lesions/number examined (percentage affected)
Nervous	Meningoencephalitis/poliomyelitis	3/3 (100%)
–	Influenza A virus detection via immunohistochemistry	3/3 (100%)
Cardiovascular	–	0/3 (0%)
Respiratory	Lipid pneumonia	1/3 (33%)
–	Pulmonary edema	1/3 (33%)
–	Pulmonary hemorrhage	1/3 (33%)
Hematolymphatic	Thymic Involution	1/3 (33%)
Digestive	Intestinal infarction (volvulus)	2/3 (66%)
Musculoskeletal	Trichinosis	1/2 (50%)
Urinary	–	0/3 (0%)
Reproductive	Active spermatogenesis	1/1 (100%)
–	Testicular hemorrhage and edema	1/1 (100%)
–	Penile bowenoid papulosis	1/1 (100%)
Hepatobiliary	–	0/3 (0%)
Integumentary	Trauma	2/2 (100%)

The second case of a polar bear confirmed to be affected by 2.3.4.4b HPAIV in Alaska was a polar bear cub (i.e., yearling) found dead on land also near Point Barrow on 9 September 2024 (Figure 1). An adult female bear, likely the mother, remained near the dead cub despite deterrence efforts; therefore, only limited external examination was initially conducted. Initial observations were dorsal recumbency, no external trauma and a distended abdomen. There was minor black fecal staining around the anus. On 13 September 2024, the adult polar bear abandoned the carcass, and it was collected for postmortem examination. Ambient low temperature at Point Barrow ranged between 2.2 and 6.1°C for the 4-day period between carcass detection and more thorough examination. The cub was female, 128 cm in body length, moderately decomposed with signs of scavenging across the belly and on the hindlimbs. Body condition was fair with a small amount of external subcutaneous fat (0.5–1 cm) and with internal fat reserves. Other gross findings included distended small intestines, twisted bowel (intestinal volvulus; >360°; clockwise), lung congestion, accumulation of fluid in the pericardium and other body cavities (sanguinal pericardial and cavitary effusion), heart dilation, and cerebral swelling and congestion. Scant gastric contents included small bird feathers and bowhead whale (*Balaena mysticetus*) tissue remnants. Observation of histopathologic lesions was limited to the nervous system (Table 1). In the brain and overlying meninges, there were signs of acute, inflammatory, and destructive processes in the central nervous system (i.e., infiltrating neutrophils and mononuclear cells and evidence of microgliosis, neuronal necrosis, neuronophagia, and vasculitis). Immunohistochemistry (IHC) on formalin-fixed paraffin-embedded (FFPE) sections of brain was used to detect influenza A virus antigen in the cytoplasm of neurons and nuclei of microglial cells in immunostained sections of the brain (Figure 2). The US Department of Agriculture National Veterinary Services Laboratories (USDA NVSL) molecularly confirmed 2.3.4.4b HPAIV in FFPE sections of the brain, though full genomic sequence information was not obtained.

**Figure 2:**
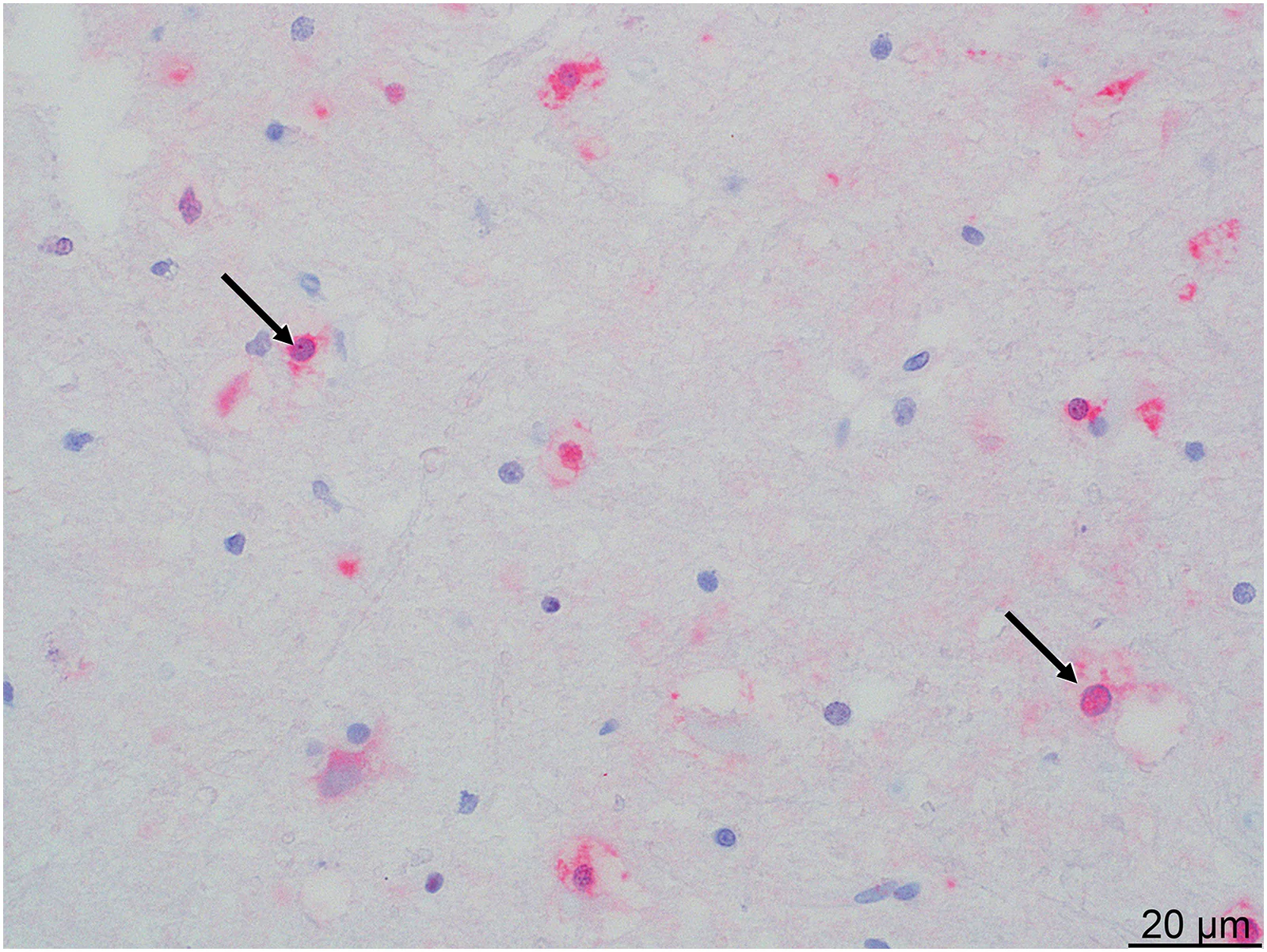
Influenza A virus antigen (indicated with arrows) in the cytoplasm of neurons and nuclei of microglial cells in immunostained sections of formalin-fixed paraffin-embedded brain tissue (shown at 40x magnification) collected from the second case of a polar bear confirmed to be affected by H5 clade 2.3.4.4b HPAIV in Alaska A 20 μm scale bar has been provided within the image for reference.

The third case of a polar bear confirmed to be affected by 2.3.4.4b HPAIV in Alaska was found dead on the beach south of Point Barrow (71.3364 °N, 156.6517 °W) on 1 August 2025 (Figure 1). The bear was adult and male, 200 cm in body length, and the carcass was moderately decomposed. Body condition was good with a substantial amount of external subcutaneous fat and with internal fat reserves. The head had been salvaged prior to carcass examination. Gross findings included two deep puncture wounds 7 cm apart on the axillary region, consistent with a bite injury from another polar bear, accompanied by muscle fascia and subcutaneous tissue necrosis extending to the chest wall, unilateral scrotal bruising with hematoma, multiple raised white cauliflower like growths (1–2 mm) present on the penile tip, distended abdomen, distended small intestines and intestinal volvulus (>360°; clockwise), lung congestion, sanguinal pericardial and cavitary effusion, and abdominal effusion admixed with blubber oil. Gastric contents were 1–2 L of rendered blubber oil. The spinal cord had liquefied due to decomposition. Histopathologic lesions were noted for the nervous, digestive, reproductive, and musculoskeletal systems (Table 1). In the spinal cord, there were signs of inflammatory and destructive processes (i.e., non-suppurative poliomyelitis with multifocal neuronal necrosis). Intestine was necrotic and hemorrhagic at the site of volvulus. Accompanying the grossly observed trauma, there was scrotal edema and hemorrhage, tearing of muscle fibers (myofiber disruption and hemorrhage), and severe inflammation of fat tissue (suppurative and hemorrhagic steatitis). The penis mass was superficial and non-invasive (a neoplasm, squamous cell carcinoma-*in situ* or bowenoid papulosis), and muscle fibers presented evidence of infection with nematodes (mild myofiber infiltration by neutrophils associated with intrasarcoplasmic *Trichinella* spp.). Influenza A virus antigen was detected in the cytoplasm of neurons and nuclei of microglial cells through IHC on FFPE sections of the spinal cord. The USDA NVSL also molecularly confirmed 2.3.4.4b HPAIV in FFPE sections of the spinal cord and from a swab of spinal tissue.

### Genomic sequences and context for HPAIVs affecting polar bears

Full-length sequences for 2.3.4.4b HPAIVs were obtained from two polar bears, the previously reported first case involving a cub found dead in 2023 and the third case identified from an adult male found dead in 2025. The virus affecting the polar bear sampled in 2023 (A/polar bear/Alaska/23–0381234/2023; GenBank: PP820319–PP820326; GISAID: EPI_ISL_18976667), a H5N1 subtype HPAIV assigned to genotype A3, was phylogenetically most closely related to contemporary 2.3.4.4b HPAIVs of the same subtype detected in wild birds in Alaska and British Columbia, Canada as inferred through analyses of the H5 and N1 gene segments (Figures 3 and S1). The virus affecting the polar bear sampled in 2025 (A/polar bear/AK/26G03248/2025; GenBank: PZ272987–PZ272994; GISAID: EPI_ISL_20423927) was confirmed as 2.3.4.4b HPAIV of the H5N5 subtype assigned to genotype A6. The H5 gene segment sequence for this virus was phylogenetically most closely related to 2.3.4.4b HPAIVs of the H5N5 subtype detected in seabirds in eastern Canada and Greenland, as well as to those detected in a northern fulmar (*Fulmarus glacialis*) and ringed seals (*Pusa hispida*) in Nunavut, Canada (Figure 3). The N5 gene segment sequence for this virus was phylogenetically most closely related to 2.3.4.4b HPAIVs of the H5N5 subtype detected in seabirds in eastern Canada and Greenland (Figure S2). Genomic sequences for 2.3.4.4b HPAIVs recovered from polar bear in 2023 and 2025 did not contain molecular markers reported to be indicative of mammalian adaptation, altered virulence, or strong drug resistance.

**Figure 3:**
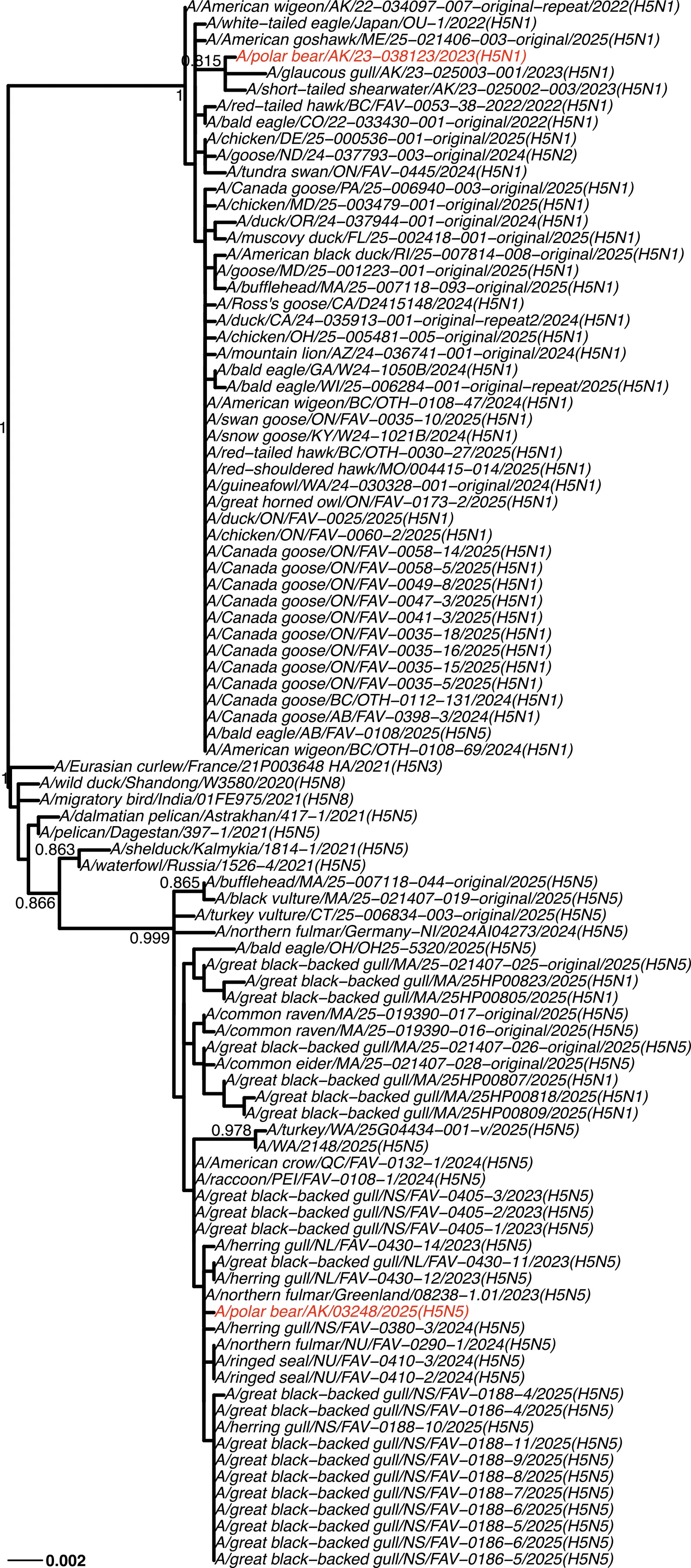
Maximum likelihood phylogeny depicting the estimated relationships among genetic sequences for H5 gene segments for clade 2.3.4.4b HPAIVs recovered from polar bears inhabiting Alaska during 2023 and 2025 (red font) and other closely related viruses from wildlife, poultry, and humans Tip labels include standard abbreviations for states within the USA and Canadian provinces. Bootstrap support values ≥ 0.700 are presented. The scale bars represent the number of substitutions per site.

### Probable non-lethal exposures of polar bears to HPAIVs

Serological assessment of exposure of two cohorts of polar bears sampled along the Arctic coast of Alaska (i.e., those sampled in 2013 and those sampled in 2023–2024) yielded markedly different results, all of which have been made publicly available in an associated data release.^24^ For 10 polar bears sampled during 2013, we found no evidence for exposure to influenza A viruses. Antibodies were not detected to the nucleoprotein (NP) through blocking enzyme-linked immunosorbent assay (bELISA), the H5 hemagglutinin gene segment through hemagglutination inhibition (HI) or microneutralization (VN), nor the N1 neuraminidase gene segment through enzyme-linked lectin assay (ELLA; Table 2). Among 14 polar bears sampled during 2023–2024, we detected antibodies to NP in sera from one bear using the manufacturer’s recommended serum/negative (S/N) ratio of <0.5 for poultry and in sera for six bears using the seropositive <0.7 threshold reported to be sensitive and specific for wild birds (Table 2).^24^ Sera from 13 of the 14 bears (93%) sampled during 2023–2024 tested positive for antibodies to the H5 clade 2.3.4.4b HPAIV antigen via HI and/or VN (Table 2).^24^ Eleven of these sera samples (79%) also tested positive for antibodies to the N1 antigen via ELLA (Table 2; Figure 1).^24^ The proportion of polar bears from which antibodies reactive to H5 clade 2.3.4.4b antigens were detected was statistically higher (α = 0.05) in the 2023–2024 cohort (13/14) as compared to the 2013 cohort (0/10) using a two-tailed Fisher’s exact test (*p* < 0.00001). Similarly, the proportion of polar bears from which antibodies reactive to H5 clade 2.3.4.4b and N1 antibodies were detected was statistically higher in the 2023–2024 cohort (11/14) as compared to 2013 cohort (0/10; Fisher’s exact test, *p* = 0.00015).

**Table 2:** Seropositivity of samples collected from polar bears inhabiting Alaska during 2013 and 2023–2024 to influenza A virus antigens

Dates of sample collection	NP bELISA+ <0.5^a^	NP bELISA+ <0.7^a^	H5 clade 2.3.4.4b+ via HI^b^	H5 clade 2.3.4.4b + via VN^c^	LPAIV H5+ via HI^b^	LPAIV H5+ via VN^c^	N1+ via ELLA^d^	Antibodies to clade 2.3.4.4b H5 and N1^e^
24 March– 26 April 2013	0/10 (0%)	0/10 (0%)	0/10 (0%)	0/10 (0%)	0/10 (0%)	0/10 (0%)	0/10 (0%)	0/10 (0%)
18 August 2023–31 August 2024	1/14 (7%)	6/14 (43%)	10/14 (71%)	13/14 (93%)	3/14 (21%)	5/14 (36%)	11/14 (79%)	11/14 (79%)

a Blocking enzyme-linked immunosorbent assay (bELISA) seropositivity (+) to the influenza A virus nucleoprotein (NP) as determined using the manufacturer’s recommendation of serum/negative optical density of <0.5 or the threshold of <0.7 evidenced to be both sensitive and specific for wild birds.

b Hemagglutination inhibition (HI) determined seropositivity (+) using a reverse genetic antigen constructed with the hemagglutinin and neuraminidase gene segments from A/Astrakhan/3212/2020 (H5N8) (H5 clade 2.3.4.4b) or/Blue-winged teal/AI12-4150/Texas/2012 (H5N2) (LPAIV H5) on PR8 backbone.

c Virus microneutralization (VN) determined seropositivity (+) using the same antigens defined in the previous footnote.^2^

d Enzyme-linked lectin assay (ELLA) determined seropositivity (+) using the antigen A/ruddy turnstone/New Jersey/AI13-2948/2013(H10N1) (N1).

e Samples testing positive for antibodies reactive to H5 (HI and/or VN) and N1 (ELLA) antigens.

Antigenic bias in seroreactivity to the H5 clade 2.3.4.4b HPAIV reagent was apparent using both HI and VN for the 11 polar bear samples that tested positive for antibodies to both H5 and N1 antigens (Table 3). That is, antibodies were more strongly reactive to the H5 clade 2.3.4.4b HPAIV antigen versus the North American low pathogenicity avian influenza virus (LPAIV) H5 reagent. For these samples, the geometric mean HI titer was 20 for the H5 clade 2.3.4.4b HPAIV antigen and 10 for the North American LPAIV H5 antigen (Table 3). A similar trend in bias was observed through VN. The geometric mean VN titer was 132 for the H5 clade 2.3.4.4b HPAIV antigen and 31 for the North American LPAIV H5 antigen (Table 3). A high geometric mean titer (530) was observed for N1 antigen among the same 11 polar bear serum samples using ELLA (Table 3).

**Table 3:** Antibody titers for 11 polar bear serum samples reactive to H5 and N1 subtype influenza A virus antigens

Sample	H5 clade 2.3.4.4b via HI^a^	LPAIV H5 via HI^a^	H5 clade 2.3.4.4b via VN^b^	LPAIV H5 via VN^b^	N1 via ELLA^c^
21832	64	8	20	<20	640
21834	64	8	80	20	1280
21835	64	16	160	<20	640
21837	16	32	320	40	640
21836	8	<8	320	<20	80
21845	16	16	320	20	640
21847	16	8	160	160	640
21848	16	8	20	1280	320
21849	16	8	320	<20	640
21419	16	8	160	<20	640
21420	<8	8	160	<20	640
**GMT**^d^	**20**	**10**	**132**	**31**	**530**

a Hemagglutination inhibition (HI) titers using a reverse genetic antigen constructed with the hemagglutinin and neuraminidase gene segments from A/Astrakhan/3212/2020 (H5N8) (H5 clade 2.3.4.4b) or/Blue-winged teal/AI12-4150/Texas/2012 (H5N2) (LPAIV H5) on PR8 backbone.

b Virus microneutralization (VN) titers using the same antigens defined in the previous footnote.^2^

c Enzyme-linked lectin assay (ELLA) titers using the antigen A/ruddy turnstone/New Jersey/AI13-2948/2013(H10N1) (N1).

d Geometric mean titers for the 11 samples indicated (in bold font).

## Discussion

In this study, we present multiple lines of evidence to suggest exposure of polar bears inhabiting land and sea-ice environments of northern Alaska during 2023–2025 to 2.3.4.4b HPAIVs with variable outcomes. The finding of three deceased bears confirmed to be infected with 2.3.4.4b HPAIVs near the community of Utqiağvik since 2023, all exhibiting evidence for neurologic disease, is consistent with these viruses emerging as biologically relevant pathogens within Arctic ecosystems. Genomic analyses suggest that two fatal HPAIV infections among polar bears did not have clear epidemiological connections, as inferred from genomic characterization of viruses, and may instead be the result of independent spillover events from wild birds or other wildlife. Though based on limited sampling, evidence of antibodies to H5 and N1 antigens among a high proportion of polar bears inhabiting coastal and nearshore habitats of Alaska during 2023–2024 indicates that contemporary non-lethal exposures to HPAIVs may be widespread among this taxon. Collectively, the data summarized herein suggest that HPAIV infection among polar bears may represent an emerging threat to polar bear health within the Alaska Arctic that will likely be challenging to control or mitigate through management actions; however, population immunity also appears to be building which may ultimately protect this species from widespread demographic impacts of disease.

Following an initial account of a polar bear cub found dead and infected with HPAIV,^13^ the formal description of two additional cases of avian influenza infection among polar bears found dead near the community of Utqiağvik in 2024 and 2025 helps to further establish 2.3.4.4b HPAIVs as emergent and biologically relevant pathogens within Arctic ecosystems and identify consistent gross and pathological signs of disease. Consistent gross and pathological findings among three cases of HPAIV infection among polar bears include meningoencephalomyelitis with vasculitis and neuronal necrosis. Therefore, 2.3.4.4b HPAIVs may exhibit neurotropism among polar bears or may be particularly virulent when exposure pathways facilitate infection of neural tissues which is consistent with reports for many other mammal species infected by 2.3.4.4b HPAIVs and presenting clinical signs or anatomical evidence of neurologic disease.^25^^,^^26^^,^^27^^,^^28^^,^^29^^,^^30^ Neurologic disease has seldom been reported in polar bears previously, and prior reports from captive animals have been attributed to other etiologies including equine herpesvirus 9, orthoreovirus, West Nile virus, and autoimmune disorders.^23^^,^^31^^,^^32^^,^^33^^,^^34^

The finding of two polar bears with distended bowels and intestinal volvulus in this study was not consistent with the previously reported case of 2.3.4.4b HPAIV in the same taxon^13^ or case reports for other bear species.^30^^,^^35^^,^^36^^,^^37^ Evidence for associated risk factors such as consumption of large quantities of food, gastrointestinal diseases, parasitism, and anatomical abnormalities,^38^^,^^39^^,^^40^ were absent in both polar bears with intestinal volvulus. Therefore, the relatedness of the observed intestinal volvulus to 2.3.4.4b HPAIV infection is indeterminate.

All three polar bears found dead and confirmed to be infected with 2.3.4.4b HPAIVs were identified in close proximity (i.e., within 15 km) of the community of Utqiağvik. Utqiağvik is the largest of relatively few communities along the sparsely populated northern Arctic coast of Alaska. Therefore, the observation of three deceased white bears near this community, located within a vast Arctic landscape that is covered by snow and ice for much of the year, may represent an observation bias rather than geographically limited extent of disease among Alaska polar bears. Lethal infections of polar bears may have occurred beyond the relatively small geographic area where carcasses were found, but mortalities were not observed.

Genomic analyses provide evidence that HPAIV infections among polar bears were of at least two combined subtypes (i.e., H5N1 and H5N5) and were introduced to polar bears through at least two epidemiologically independent pathways. The detection of a H5N1 HPAIV of the A3 genotype in 2023 is spatiotemporally consistent with other viral detections made among wildlife in Alaska through active and passive surveillance.^41^ Viruses of this subtype and specific genotype were previously reported to be introduced by wild birds into Alaska from East Asia through the overlapping East Asian-Australasian and Pacific Americas flyways where they have been maintained among wild birds inhabiting this region since at least early 2022.^42^ In contrast, the detection of a H5N5 genotype A6 virus from a deceased polar bear sampled in 2025 represents the first report of a 2.3.4.4b HPAIV of this subtype and genotype within Alaska. Curiously, a 2.3.4.4b HPAIV was recently reported from a deceased polar bear identified in Svalbard, Norway.^43^ Previously, H5N5 genotype A6 HPAIVs were reported to have been introduced into northeastern North America from Europe purportedly via seabirds, circa 2023 where they subsequently affected diverse wild birds and mammals.^44^ These H5N5 2.3.4.4b HPAIVs appear to have spread westward, sporadically developing mutations conferring mammalian adaptation.^44^^,^^45^ Phylogenetic analyses presented herein are generally consistent with previously described routes of introduction and viral evolution, though, they also support more extensive spatiotemporal viral dispersal via North American wildlife than previously reported. Collectively, these data provide evidence that HPAIV infections among polar bears have been the result of at least two independent spillover events through unknown exposure pathways, though wild birds or other unidentified wildlife hosts were likely involved. Further research would be needed to determine if infections among polar bears may be the result of scavenging of bird carcasses, consumption of ice seal prey previously unrecognized to be infected with HPAIVs in Alaska, or other unidentified environmental exposure routes.

Though based on limited sampling, the finding that most apparently healthy polar bears live-captured along the Arctic coast of Alaska during 2023–2024 had antibodies reactive to a 2.3.4.4b H5 antigen provides evidence that contemporary non-lethal exposure to HPAIVs may be extensive among this taxon. The additional findings that most of these bears also had antibodies reactive to an N1 antigen, that geometric mean titers supported antigenic bias toward the 2.3.4.4b H5 antigen versus the LPAIV H5 antigen in serologic assays, and that this study and previous research identified no evidence for influenza exposure among polar bears sampled in Alaska prior to 2023,^21^ are all also consistent with contemporary exposure of polar bears to 2.3.4.4b HPAIVs. Recent results from Norway further corroborate the premise that contemporaneous exposures to 2.3.4.4b HPAIVs may be widespread among polar bears inhabiting the Arctic with variable outcomes.^43^ The factors contributing to variable outcomes for HPAIV infection among polar bears are unclear, though pathogenesis may be influenced by route and dose of exposure, individual immune response, and the specific characteristics of the virus to which an individual is exposed.^46^^,^^47^

The finding of a high proportion of live-captured polar bears with antibodies reactive to 2.3.4.4b H5 and N1 antigens is consistent with previous research that found large proportions of brown bears (*Ursus arctos*) and red fox (*Vulpes vulpes*) sampled within the Alaska Arctic during December 2021–September 2023 to be seropositive for the same 2.3.4.4b H5 and N1 antigens.^48^ Another investigation previously reported numerous 2.3.4.4b HPAIV infections among wild birds inhabiting the Alaska Arctic during 2022.^41^ Collectively, these results indicate that HPAIV infection may be widespread not only among specific taxa, but also within Arctic Alaska ecosystems and food webs. Curiously, the bELISA for the NP antigen did not appear to be a particularly sensitive screening tool for identifying prior H5 and N1 avian influenza virus exposure among polar bears, which was also true among Alaska brown bears, although perhaps to a lesser extent.^49^ The underlying reasons why the bELISA did not identify NP seropositivity among bears exhibiting strong serologic responses to H5 clade 2.3.4.4b HPAIV and N1 antigens are unknown, but point to the importance of utilizing multiple data streams to derive inference when applying assays to non-model species.

Given the mounting evidence that HPAIV exposure may now be widespread among wildlife inhabiting the Alaska Arctic, including polar bears using coastal and nearshore habitats, cost-effective, scalable, and effective management actions to control viral spread or mitigate disease within the broader region would likely be challenging. Vaccination programs have been developed and applied for other viral diseases among wildlife with success in specific contexts (e.g., rabies^50^), but comparable measures for HPAIVs are still in very early stages of development and have not yet been demonstrated to be effective and deployable at scale.^51^^,^^52^^,^^53^ As such, management agencies may have few options for directly addressing HPAIV or mitigating impacts thereof in the short-term and may choose to instead assess other limited options to help foster wildlife health and promote resiliency. Data summarized herein provide evidence that not all 2.3.4.4b HPAIV exposures are fatal among polar bears, indicating that population immunity could ultimately protect this species from widespread demographic impacts of disease.

### Limitations of this study

Given the low density of abundance of polar bears as well as logistical, cultural, financial, and regulatory considerations associated with capture and handling efforts for this species, sampling for the research summarized herein was limited and spatiotemporally biased within the Alaska Arctic. Discussion of results has therefore been qualified to acknowledge these constraints.

## Resource availability

### Lead contact

Requests for additional information or resources should be directed to and will be fulfilled by the lead contact, Andrew M. Ramey (aramey@usgs.gov).

### Materials availability

The authors will make materials available to requesting parties, as feasible, which may require an institutional material transfer agreement.

### Data and code availability

• Viral genomic sequence data have been deposited at GenBank (PP820319–PP820326 and PZ272987–PZ272994), and GISAID (accession numbers EPI_ISL_18976667 and EPI_ISL_20423927), and are publicly available as of the date of publication. Accession numbers are also listed in the key resources table. Live-animal capture and accompanying serologic data have been deposited at Science Base (https://doi.org/10.5066/P13WGATT) and are publicly available as of the date of publication. The web address to access data is also provided in the key resources table.
• No original code was generated as part of this study.
• The authors will make additional materials available to requesting parties, as feasible, which may require an institutional material transfer agreement.

## Acknowledgments

We thank the numerous individuals that helped to collect or analyze samples for this project including Andy Von Duyke, Anthony Pagano, Chris Michetti, Dan Bjornlie, Dave Gustine, Deborah Carter, Eric Van Dongen, Erik Andersen, Erin Earhart, Jennifer Dushane, Mike Sandstrom, Riley Wilson, Ryan Wilson, Susannah Woodruff, and Zijing Cao. We appreciate the review of serologic data underlying this summary provided by Justin Brown. We appreciate constructive critiques on prior versions of this manuscript provided by Andrew Lang and three anonymous reviewers. Any use of trade, firm, or product names is for descriptive purposes only and does not imply endorsement by the US government. This project was funded, in part, by the US Geological Survey through the Biological Threats and Invasive Species Research program of the Ecosystems Mission Area and the 10.13039/100000060National Institute of Allergy and Infectious Diseases, National Institutes of Health, Department of Health and Human Services (contract no. 75N93021C00016).

## Author contributions

A.M.R.: conceptualization, data curation, formal analysis, funding acquisition, investigation, methodology, project administration, resources, writing – original draft, and writing – review and editing; L.S.M.: conceptualization, data curation, funding acquisition, investigation, resources, writing – original draft, and writing – review and editing; T.C.A.: data curation, formal analysis, funding acquisition, investigation, resources, visualization, writing – original draft, and writing – review and editing; R.S.: data curation, formal analysis, funding acquisition, investigation, methodology, resources, writing – original draft, and writing – review and editing; D.S.R.: data curation, formal analysis, investigation, methodology, resources, visualization, writing – original draft, and writing – review and editing; C.A.A.: data curation, formal analysis, investigation, methodology, visualization, writing – original draft, and writing – review and editing; K.S.: data curation, investigation, and writing – review and editing; S.M.C.: data curation, investigation, resources, and writing – review and editing; M.K.T.: data curation, investigation, resources, and writing – review and editing; D.E.S.: conceptualization, data curation, formal analysis, funding acquisition, investigation, methodology, resources, validation, writing – original draft, and writing – review and editing; R.L.P.: conceptualization, data curation, formal analysis, funding acquisition, investigation, methodology, project administration, resources, validation, writing – original draft, and writing – review and editing.

## Declaration of interests

The authors declare no competing interests.

## STAR★Methods

### Key resources table

**Table:** 

REAGENT or RESOURCE	SOURCE	IDENTIFIER
**Bacterial and virus strains**		

influenza A virus antigen	Abcam	Cat#ab20841; RRID: AB_775660

**Chemicals, peptides, and recombinant proteins**		

Telazol®	Fort Dodge Animal Health, Warner-Lambert Co.	National Drug Code: 54771–9050-01

**Critical commercial assays**		

AI MultiS-Screen Ab test	IDEXX Laboratories	Cat#99–12119

**Deposited data**		

Viral genomic sequence data	This study	GenBank: PP820319–PP820326 and PZ272987– PZ272994
Viral genomic sequence data	This study	GISAID: EPI_ISL_18976667 and EPI_ISL_20423927
Live-animal capture data	This study	ScienceBase: https://doi.org/10.5066/P13WGATT
Serologic data	This study	ScienceBase: https://doi.org/10.5066/P13WGATT

**Software and algorithms**		

R	R Core Team^54^	https://cran.r-project.org/doc/manuals/r-release/fullrefman.pdf
DECIPHER	Wright^55^	https://journal.r-project.org/articles/RJ-2024-022/
Phangorn	Schliep^56^	https://klausvigo.github.io/phangorn/
FluSurver	GISAID	https://gisaid.org/database-features/flusurver-mutations-app/

**Other**		

DNA/RNA shield media	Zymo	Cat#R1100-50

### Experimental model and study participant details

Serology was used to infer prior non-lethal exposure of polar bears to influenza A viruses, including 2.3.4.4b HPAIVs. We tested sera from two cohorts of bears: (1) those sampled during 2013, prior to the first incursion of H5 subtype HPAIVs into North America by wild birds^57^ and (2) those sampled during 2023–2024 following more recent incursions of 2.3.4.4b HPAIVs into North America and dissemination throughout Alaska.^41^ Sera were collected from apparently healthy, free-ranging polar bears inhabiting Alaska that had been captured as part of federal research and monitoring programs in the USA as authorized through Marine Mammal Protection Act and Endangered Species Act research permit MA82088B-1, U.S. Fish and Wildlife Service IACUC #2023-001, Marine Mammal Permit 690038, and USGS Animal Care and Use Committee permits 2010–2014 and 2023–2025. Captured animals included both male (*n* = 8) and female (*n* = 16) bears as well as adult (*n* = 18) and sub-adult (*n* = 6) animals. Bears were either captured during March and April on sea ice within approximately 100 km of the Arctic coast of Alaska’s southern Beaufort Sea between Utqiağvik and the USA-Canada border (*n* = 13) or within coastal terrestrial habitats near Prudhoe Bay during August and September (*n* = 11). Bears were captured on sea ice using a helicopter and captured on land with aluminum culvert traps. Bears were immobilized with tiletamine hydrochloride plus zolazepam hydrochloride (Telazol, Fort Dodge Animal Health, Fort Dodge, Iowa, USA, and Warner-Lambert Co., Groton, Connecticut, USA) using projectile syringes fired from a dart gun or administered via a pole syringe. All bears were fitted with plastic ear-tags, weighed, and measured. Bears captured on land also received passive integrated transponders, glue-on transmitters (SPLASH10-BF-297O, Wildlife Computers, Redmond, WA), and Iridium-enabled GPS collars (Telonics Inc., Mesa, Arizona, USA) with automatic release devices (adult females only). Blood was collected from all bears via venipuncture of either a femoral or jugular vein. Blood samples were centrifuged to separate sera from cellular components. Sera was decanted, frozen, and sent to the Southeastern Cooperative Wildlife Disease Study (SCWDS) at the University of Georgia where it was stored at −20°C for serologic testing.

### Method details

#### Case reports

Sightings of all polar bears found dead and confirmed to be infected with 2.3.4.4b HPAIVs were reported by community members of Utqiağvik while traveling on the land. Postmortem examinations were conducted by the North Slope Borough Division of Wildlife Management wildlife veterinarian. Case reports were generated by describing gross lesions observed during postmortem examination as well as histopathological and immunohistochemical findings. Collection of polar bear tissues occurred under USFWS permit # MA80164B issued to the North Slope Borough Department of Wildlife Management.

Tissue samples collected from polar bears were fixed for a minimum of 2 weeks in 10% neutral buffered formalin and processed for routine histopathological examination with hematoxylin and eosin staining (Histology Consultation Services, Everson, Washington). Tissues examined included brain (cases 1–2), spinal cord (case 3), liver (cases 1–3), lung (cases 1–3), kidney (case 1 and 3), lymph node and/or thymus (cases 1–3), spleen (cases 1–2), heart (cases 1–3), skeletal muscle (cases 1–3), small intestine (cases 1–3), testis (case 3), and penis (case 3). IHC was performed on FFPE sections of brain (*n* = 2) or spinal cord (*n* = 1) to detect influenza A virus antigen (polyclonal, catalog number ab20841, Abcam, Waltham, Massachusetts, USA) at the Athens Disease Diagnostic Laboratory at the University of Georgia. Scrolls of the FFPE cerebrum (*n* = 2) and spinal cord (*n* = 1) were sent to the USDA NVSL for molecular testing and confirmation. For case 3, spinal cord, rectal, and muscle swabs were collected in DNA/RNA shield media (Zymo, Irvine, California, USA) and stored frozen until they were analyzed. Swabs were sent to the USDA NVSL for molecular testing and confirmation. Molecular testing, confirmation, sequencing, and genotyping of 2.3.4.4b HPAIVs followed previously reported procedures.^4^

#### Genomic analyses

Full-length sequences for HPAIVs originating from samples collected from polar bears in 2023 (brain tissue) and 2025 (spinal swab) were obtained and genotyped using previously reported methods.^4^ Sequences for the H5, N1, and N5 gene segments were compared to HPAIVs from other wild birds, mammals, poultry, and humans as follows. Sequences for the H5, N1, and N5 gene segments of polar bear 2023 and 2025 HPAIVs were first compared to those on the GISAID (https://gisaid.org/) and GenBank (https://www.ncbi.nlm.nih.gov/genbank/) databases using local BLAST functions.^58^^,^^59^ The top 25 sequences sharing highest nucleotide identity for each GISAID and GenBank BLAST searches were downloaded, collated, and duplicate sequences removed. Alignments were next created for each gene segment using the package ‘DECIPHER’^55^ in R.^54^ The R package ‘phangorn’^56^ was then used to generate midpoint rooted maximum likelihood phylogenetic trees using the nucleotide substitution model with the lowest AICc and 1000 bootstrap replicates. Viral dissemination was inferred using the phylogenetic position of sequences for HPAIVs originating from samples collected from polar bears within clades of closely related viruses. Molecular markers potentially indicative of mammalian adaptation, altered virulence, or strong drug resistance of HPAIVs were assessed from the complete coding regions of relevant gene segments using FluSurver (https://gisaid.org/database-features/flusurver-mutations-app/).

#### Serology

With minor modifications, serology followed procedures previously applied for assessing exposure of wild mammals inhabiting Alaska to 2.3.4.4b HPAIVs.^48^ Briefly, serum samples were heat inactivated at 57°C and then tested in duplicate for antibodies to the influenza A virus NP by using a commercially available bELISA, specifically the AI MultiS-Screen Ab test, (IDEXX Laboratories, Westbrook, Maine, USA; https://www.idexx.com), at SCWDS. Positivity was assessed using the average S/N optical density ratio for duplicate runs using both the manufacturer’s recommended threshold for poultry (<0.5) and the limit of detection evidenced to be both sensitive and specific for wild birds (<0.7).^60^^,^^61^ All sera samples were then tested using HI and VN for antibodies to 2.3.4.4b HPAIV and North American lineage H5 LPAIV using a reverse genetic antigen (IDCDC-RG71A [H5N8]) constructed with the hemagglutinin and neuraminidase gene segments from A/Astrakhan/3212/2020 (H5N8) on PR8 backbone or a reverse genetic antigen constructed with the hemagglutinin and neuraminidase gene segments from A/Blue-winged teal/AI12-4150/Texas/2012 (H5N2) on PR8 backbone per previously described procedures.^62^^,^^63^ All sera samples were also tested for reactivity to N1 subtype influenza A viruses by using ELLA utilizing the antigen A/ruddy turnstone/New Jersey/AI13-2948/2013(H10N1) as previously reported.^63^ Seropositivity was determined on the basis of HI (≥16), VN (≥20), and ELLA (≥80) titers.

### Quantification and statistical analysis

Serologic data were used to test for statistical differences in the detection of antibodies reactive to 2.3.4.4b HPAIV-associated antibodies among two previously defined cohorts of polar bears using two-tailed Fisher’s exact tests in R.^54^ An initial Fisher’s exact test considered differences among the detection of antibodies reactive to H5 clade 2.3.4.4b antibodies via HI and/or VN among bears sampled during 2013 versus those sampled during 2023–2024. A second Fisher’s exact test considered differences among the detection of antibodies reactive to H5 clade 2.3.4.4b antibodies via HI and/or VN and N1 antibodies via ELLA among bears sampled during 2013 versus those sampled during 2023–2024. *p*-values <0.05 were considered statistically significant.

## References

[1] Ramey A.M., Hill N.J., DeLiberto T.J., Gibbs S.E.J., Camille Hopkins M., Lang A.S., Poulson R.L., Prosser D.J., Sleeman J.M., Stallknecht D.E., Wan X. (2022). Highly pathogenic avian influenza is an emerging disease threat to wild birds in North America. J. Wildl. Manag..

[2] Leguia M., Garcia-Glaessner A., Muñoz-Saavedra B., Juarez D., Barrera P., Calvo-Mac C., Jara J., Silva W., Ploog K., Amaro L. (2023). Highly pathogenic avian influenza A (H5N1) in marine mammals and seabirds in Peru. Nat. Commun..

[3] Puryear W., Sawatzki K., Hill N., Foss A., Stone J.J., Doughty L., Walk D., Gilbert K., Murray M., Cox E. (2023). Highly pathogenic avian influenza A (H5N1) virus outbreak in New England seals, United States. Emerg. Infect. Dis..

[4] Youk S., Torchetti M.K., Lantz K., Lenoch J.B., Killian M.L., Leyson C., Bevins S.N., Dilione K., Ip H.S., Stallknecht D.E. (2023). H5N1 highly pathogenic avian influenza clade 2.3.4.4b in wild and domestic birds: introductions into the United States and reassortments, December 2021–April 2022. Virology.

[5] Caserta L.C., Frye E.A., Butt S.L., Laverack M., Nooruzzaman M., Covaleda L.M., Thompson A.C., Koscielny M.P., Cronk B., Johnson A. (2024). Spillover of highly pathogenic avian influenza H5N1 virus to dairy cattle. Nature.

[6] Garg S., Reed C., Davis C.T., Uyeki T.M., Behravesh C.B., Kniss K., Budd A., Biggerstaff M., Adjemian J., Barnes J.R. (2024). Outbreak of highly pathogenic avian influenza A (H5N1) viruses in US dairy cattle and detection of two human cases—United States, 2024. MMWR Morb. Mortal. Wkly. Rep..

[7] Plaza P.I., Gamarra-Toledo V., Euguí J.R., Lambertucci S.A. (2024). Recent changes in patterns of mammal infection with highly pathogenic avian influenza A (H5N1) virus worldwide. Emerg. Infect. Dis..

[8] Webby R.J., Uyeki T.M. (2024). An update on highly pathogenic avian influenza A (H5N1) virus, clade 2.3.4.4b. J. Infect. Dis..

[9] Caliendo V., Lewis N.S., Pohlmann A., Baillie S.R., Banyard A.C., Beer M., Brown I.H., Fouchier R.A.M., Hansen R.D.E., Lameris T.K. (2022). Transatlantic spread of highly pathogenic avian influenza H5N1 by wild birds from Europe to North America in 2021. Sci. Rep..

[10] Banyard A.C., Bennison A., Byrne A.M.P., Reid S.M., Lynton-Jenkins J.G., Mollett B., De Silva D., Peers-Dent J., Finlayson K., Hall R. (2024). Detection and spread of high pathogenicity avian influenza virus H5N1 in the Antarctic Region. Nat. Commun..

[11] Clessin A., Briand F.X., Tornos J., Lejeune M., De Pasquale C., Fischer R., Souchaud F., Hirchaud E., Hong S.L., Bralet T. (2025). Circumpolar spread of avian influenza H5N1 to southern Indian Ocean islands. Nat. Commun..

[12] Daniels B.L., Osnas E.E., Boldenow M.L., Gerlach R.F., Ahlstrom C.A., Coburn S.M., Brook M.J., Brubaker M., Fischer J.B., Koons D.N. (2025). Observational, virologic, and serologic data provide insights into an outbreak of highly pathogenic avian influenza among wild birds on the Yukon-Kuskokwim Delta, Alaska, USA, in 2022. J. Wildl. Dis..

[13] Stimmelmayr R., Rotstein D., Torchetti M.K., Gerlach R. (2024). Highly pathogenic avian influenza virus A (H5N1) clade 2.3.4.4b infection in free-ranging polar bear, Alaska, USA. Emerg. Infect. Dis..

[14] Pagano A.M., Davis R.W., Pagano A.M. (2021). Ethology and Behavioral Ecology of Sea Otters and Polar Bears.

[15] Stimmelmayr R., SimsKayotuk C., Pederson M., Sheffield G., Frantz R., Nayakik J., Adams B. (2023). Anthropogenic waste ingestion of Southern Beaufort Sea polar bears, Alaska (2010–2020). Ursus.

[16] Rode K.D., Wilson R.R., Regehr E.V., St Martin M., Douglas D.C., Olson J. (2015). Increased land use by Chukchi Sea polar bears in relation to changing sea ice conditions. PLoS One.

[17] Atwood T.C., Peacock E., McKinney M.A., Lillie K., Wilson R., Douglas D.C., Miller S., Terletzky P. (2016). Rapid environmental change drives increased land use by an Arctic marine predator. PLoS One.

[18] Atwood T.C., Duncan C., Patyk K.A., Nol P., Rhyan J., McCollum M., McKinney M.A., Ramey A.M., Cerqueira-Cézar C.K., Kwok O.C.H. (2017). Environmental and behavioral changes may influence the exposure of an Arctic apex predator to pathogens and contaminants. Sci. Rep..

[19] Pilfold N.W., Richardson E.S., Ellis J., Jenkins E., Scandrett W.B., Hernández-Ortiz A., Buhler K., McGeachy D., Al-Adhami B., Konecsni K. (2021). Long-term increases in pathogen seroprevalence in polar bears (*Ursus maritimus*) influenced by climate change. Glob. Change Biol..

[20] Rode K.D., Van Hemert C., Wilson R.R., Woodruff S.P., Pabilonia K., Ballweber L., Kwok O., Dubey J.P. (2024). Increased pathogen exposure of a marine apex predator over three decades. PLoS One.

[21] Hemert C.V., Spivey T.J., Uher-Koch B.D., Atwood T.C., Sinnett D.R., Meixell B.W., Hupp J.W., Jiang K., Adams L.G., Gustine D.D. (2019). Survey of Arctic Alaskan wildlife for influenza A antibodies: limited evidence for exposure of mammals. J. Wildl. Dis..

[22] Naidenko S.V., Ivanov E.A., Mordvintsev I.N., Platonov N.G., Ershov R.V., Rozhnov V.V. (2013). Seropositivity for different pathogens in polar bears (*Ursus maritimus*) from Barents Sea islands. Biol. Bull..

[23] Szentiks C.A., Tsangaras K., Abendroth B., Scheuch M., Stenglein M.D., Wohlsein P., Heeger F., Höveler R., Chen W., Sun W. (2014). Polar bear encephalitis: establishment of a comprehensive next-generation pathogen analysis pipeline for captive and free-living wildlife. J. Comp. Pathol..

[24] Ramey A.M., Mangipane L.S., Atwood T.C., Stimmelmayr R., Rotstein D., Ahlstrom C.A., Simac K., Coburn S., Kim Torchetti M., Stallknecht D.E., Poulson R.L. (2026). Data on exposure of polar bears inhabiting Alaska to highly pathogenic H5 clade 2.3.4.4b and N1 avian influenza viruses. U.S. Geological Survey data release.

[25] Elsmo E.J., Wünschmann A., Beckmen K.B., Broughton-Neiswanger L.E., Buckles E.L., Ellis J., Fitzgerald S.D., Gerlach R., Hawkins S., Ip H.S. (2023). Highly pathogenic avian influenza A (H5N1) virus clade 2.3.4.4b infections in wild terrestrial mammals, United States, 2022. Emerg. Infect. Dis..

[26] Alkie T.N., Cox S., Embury-Hyatt C., Stevens B., Pople N., Pybus M.J., Xu W., Hisanaga T., Suderman M., Koziuk J. (2023). Characterization of neurotropic HPAI H5N1 viruses with novel genome constellations and mammalian adaptive mutations in free-living mesocarnivores in Canada. Emerg. Microb. Infect..

[27] Bordes L., Vreman S., Heutink R., Roose M., Venema S., Pritz-Verschuren S.B.E., Rijks J.M., Gonzales J.L., Germeraad E.A., Engelsma M., Beerens N. (2023). Highly pathogenic avian influenza H5N1 virus infections in wild red foxes (*Vulpes vulpes*) show neurotropism and adaptive virus mutations. Microbiol. Spectr..

[28] Vreman S., Kik M., Germeraad E., Heutink R., Harders F., Spierenburg M., Engelsma M., Rijks J., van den Brand J., Beerens N. (2023). Zoonotic mutation of highly pathogenic avian influenza H5N1 virus identified in the brain of multiple wild carnivore species. Pathogens.

[29] Lair S., Quesnel L., Signore A.V., Delnatte P., Embury-Hyatt C., Nadeau M.S., Lung O., Ferrell S.T., Michaud R., Berhane Y. (2024). Outbreak of highly pathogenic avian influenza A (H5N1) virus in seals, St. Lawrence Estuary, Quebec, Canada. Emerg. Infect. Dis..

[30] Beckmen K.B., Burek Huntington K.A., Spraker T., Ellis J. (2025). Pathologic characterization of highly pathogenic H5N1 avian influenza virus infections in wild mammals in Alaska, USA. J. Wildl. Dis..

[31] Kenny D.E., Baier J., Knightly F., Steinheimer D., Getzy D.M., Shelton G.D. (2004). Myasthenia gravis in a polar bear (*Ursus maritimus*). J. Zoo Wildl. Med..

[32] Donovan T.A., Schrenzel M.D., Tucker T., Pessier A.P., Bicknese B., Busch M.D.M., Wise A.G., Maes R., Kiupel M., McKnight C., Nordhausen R.W. (2009). Meningoencephalitis in a polar bear caused by equine herpesvirus 9 (EHV-9). Vet. Pathol..

[33] Dutton C.J., Quinnell M., Lindsay R., DeLay J., Barker I.K. (2009). Paraparesis in a polar bear (*Ursus maritimus*) associated with West Nile virus infection. J. Zoo Wildl. Med..

[34] Prüss H., Leubner J., Wenke N.K., Czirják G.Á., Szentiks C.A., Greenwood A.D. (2015). Anti-NMDA receptor encephalitis in the polar bear (*Ursus maritimus*) Knut. Sci. Rep..

[35] Bessière P., Gaide N., Croville G., Crispo M., Fusade-Boyer M., Abou Monsef Y., Dirat M., Beltrame M., Dendauw P., Lemberger K. (2024). High pathogenicity avian influenza A (H5N1) clade 2.3.4.4b virus infection in a captive Tibetan black bear (*Ursus thibetanus*): investigations based on paraffin-embedded tissues, France, 2022. Microbiol. Spectr..

[36] Jakobek B.T., Berhane Y., Nadeau M.S., Embury-Hyatt C., Lung O., Xu W., Lair S. (2023). Influenza A (H5N1) virus infections in 2 free-ranging black bears (*Ursus americanus*), Quebec, Canada. Emerg. Infect. Dis..

[37] Briand F.X., Bessière P., Gaide N., Croville G., Crispo M., Fusade-Boyer M., Abou Monsef Y., Dirat M., Beltrame M., Dendauw P., Lemberger K., Guérin J.L. (2024). Highly pathogenic avian influenza A (H5N1) clade 2.3.4.4b virus infection in captive bears (*Ursus thibetanus*) and in captive and wild birds, France, 2022. German Journal of Veterinary Research.

[38] Begeman L., St Leger J.A., Blyde D.J., Jauniaux T.P., Lair S., Lovewell G., Raverty S., Seibel H., Siebert U., Staggs S.L. (2013). Intestinal volvulus in cetaceans. Vet. Pathol..

[39] Rami Reddy S.R., Cappell M.S. (2017). A systematic review of the clinical presentation, diagnosis, and treatment of small bowel obstruction. Curr. Gastroenterol. Rep..

[40] Ludes-Wehrmeister E., Wohlsein P., Prenger-Berninghoff E., Ewers C., Woelfing B., Lehnert K., Siebert U. (2020). Intestinal displacements in older harbour and grey seals. Dis. Aquat. Org..

[41] Ahlstrom C.A., Torchetti M.K., Lenoch J., Beckmen K., Boldenow M., Buck E.J., Daniels B., Dilione K., Gerlach R., Lantz K. (2024). Genomic characterization of highly pathogenic H5 avian influenza viruses from Alaska during 2022 provides evidence for genotype-specific trends of spatiotemporal and interspecies dissemination. Emerg. Microb. Infect..

[42] Ramey A.M., Scott L.C., Ahlstrom C.A., Buck E.J., Williams A.R., Kim Torchetti M., Stallknecht D.E., Poulson R.L. (2024). Molecular detection and characterization of highly pathogenic H5N1 clade 2.3.4.4b avian influenza viruses among hunter-harvested wild birds provides evidence for three independent introductions into Alaska. Virology.

[43] Madslien K., Fosse J.H., Aars J., Bøe C.A., Andersen M., Buhler K., Fjeldheim I., Gjerset B., Jørgensen T., Myhrvold I.K., Rohringer A. (2026). Highly pathogenic avian influenza H5N5 in a polar bear and Atlantic walrus, Svalbard, 2026, with widespread seroconversion in polar bears. bioRxiv.

[44] Erdelyan C.N.G., Kandeil A., Signore A.V., Jones M.E.B., Vogel P., Andreev K., Bøe C.A., Gjerset B., Alkie T.N., Yason C. (2024). Multiple transatlantic incursions of highly pathogenic avian influenza clade 2.3.4.4b A (H5N5) virus into North America and spillover to mammals. Cell Rep..

[45] Rahman I., Erdelyan C.N.G., Signore A.V., Ayilara I., Wight J., Jones M.E.B., Sullivan D.S., Lung O., Hisanaga T., Wilhelm S.I. (2024). Recurring incursions and dissemination of novel Eurasian-origin H5Nx avian influenza viruses in Atlantic Canada. Virus Evol..

[46] Fukuyama S., Kawaoka Y. (2011). The pathogenesis of influenza virus infections: the contributions of virus and host factors. Curr. Opin. Immunol..

[47] Bauer L., Benavides F.F.W., Veldhuis Kroeze E.J.B., de Wit E., Van Riel D. (2023). The neuropathogenesis of highly pathogenic avian influenza H5Nx viruses in mammalian species including humans. Trends Neurosci..

[48] Ramey A.M., Beckmen K.B., Saalfeld D.T., Nicholson K.L., Mangipane B.A., Scott L.C., Stallknecht D.E., Poulson R.L. (2025). Exposure of wild mammals to influenza A (H5N1) virus, Alaska, USA, 2020–2023. Emerg. Infect. Dis..

[49] Scott L.C., Ramey A.M., Beckmen K.B., Saafeld D.T., Nicholson K.L., Mangipane B.A., Stallknecht D.E., Poulson R.L. (2024). https://www.usgs.gov/data/data-exposure-wild-mammals-inhabiting-alaska-h5n1-influenza-a-viruses.

[50] Maki J., Guiot A.L., Aubert M., Brochier B., Cliquet F., Hanlon C.A., King R., Oertli E.H., Rupprecht C.E., Schumacher C. (2017). Oral vaccination of wildlife using a vaccinia–rabies-glycoprotein recombinant virus vaccine (RABORAL V-RG®): a global review. Vet. Res..

[51] Roberts L.C., Abernethy D., Roberts D.G., Ludynia K., O’Kennedy M.M., Abolnik C. (2023). Vaccination of African penguins (*Spheniscus demersus*) against high-pathogenicity avian influenza. Vet. Rec..

[52] Katzner T.E., Blackford A.V., Donahue M., Gibbs S.E.J., Lenoch J., Martin M., Rocke T.E., Root J.J., Styles D., Cooper S. (2025). Safety and immunogenicity of poultry vaccine for protecting critically endangered avian species against highly pathogenic avian influenza virus, United States. Emerg. Infect. Dis..

[53] Lejeune M., Tornos J., Bralet T., De Pasquale C., Marçon E., Massin P., Grasland B., Stier A., Boulinier T. (2026). Vaccination against H5 HP avian influenza virus leads to persistent immune response in wild king penguins. Nat. Commun..

[54] R Core Team (2016).

[55] Wright E.S. (2024). Fast and Flexible Search for Homologous Biological Sequences with DECIPHER v3. RELC J..

[56] Schliep K.P. (2011). phangorn: phylogenetic analysis in R. Bioinformatics.

[57] Ip H.S., Torchetti M.K., Crespo R., Kohrs P., DeBruyn P., Mansfield K.G., Baszler T., Badcoe L., Bodenstein B., Shearn-Bochsler V. (2015). Novel Eurasian highly pathogenic avian influenza A H5 viruses in wild birds, Washington, USA, 2014. Emerg. Infect. Dis..

[58] Johnson M., Zaretskaya I., Raytselis Y., Merezhuk Y., McGinnis S., Madden T.L. (2008). NCBI BLAST: a better web interface. Nucleic Acids Res..

[59] Shu Y., McCauley J. (2017). GISAID: from vision to reality. Euro Surveill..

[60] Brown J.D., Luttrell M.P., Berghaus R.D., Kistler W., Keeler S.P., Howey A., Wilcox B., Hall J., Niles L., Dey A. (2010). Prevalence of antibodies to type A influenza virus in wild avian species using two serologic assays. J. Wildl. Dis..

[61] Shriner S.A., Van Dalen K.K., Root J.J., Sullivan H.J. (2016). Evaluation and optimization of a commercial blocking ELISA for detecting antibodies to influenza A virus for research and surveillance of mallards. J. Virol. Methods.

[62] Stallknecht D.E., Fojtik A., Carter D.L., Crum-Bradley J.A., Perez D.R., Poulson R.L. (2022). Naturally acquired antibodies to influenza A virus in fall-migrating North American mallards. Vet. Sci..

[63] Stallknecht D.E., Kienzle-Dean C., Davis-Fields N., Jennelle C.S., Bowman A.S., Nolting J.M., Boyce W.M., Crum J.M., Santos J.J.S., Brown J.D. (2020). Limited detection of antibodies to clade 2.3.4.4 A/goose/Guangdong/1/1996 lineage highly pathogenic H5 avian influenza virus in North American waterfowl. J. Wildl. Dis..

