# Supplementary material for: Evidence for exposure to and infection with H5 clade 2.3.4.4b highly pathogenic avian influenza viruses among polar bears inhabiting Alaska
Source: iScience. 2026 Sep 22;29(10):117736. doi: 10.1016/j.isci.2026.117736 (PMC13673100; doi:10.1016/j.isci.2026.117736)

iScience, Volume *29*

Supplemental information

Evidence for exposure to and infection with H5 clade 2.3.4.4b highly pathogenic avian influenza viruses among polar bears inhabiting Alaska

Andrew M. Ramey, Lindsey S. Mangipane, Todd C. Atwood, Raphaela Stimmelmayr, David S. Rotstein, Christina A. Ahlstrom, Kristin Simac, Sarah M. Coburn, Mia Kim Torchetti, David E. Stallknecht, and Rebecca L. Poulson

Supplemental figures for 'Evidence for exposure to and infection with H5 clade 2.3.4.4b highly pathogenic avian influenza viruses among polar bears inhabiting Alaska'

Andrew M. Ramey[1]*, Lindsey S. Mangipane[2], Todd C. Atwood[1], Raphaela Stimmelmayr[3,4], David S. Rotstein[5], Christina A. Ahlstrom[1], Kristin Simac[1], Sarah M. Coburn[6], Mia Kim Torchetti[7], David E. Stallknecht[8], Rebecca L. Poulson[8]

[1]U.S. Geological Survey, Alaska Science Center, Anchorage, Alaska, USA

[2]U.S. Fish and Wildlife Service, Marine Mammals Management, Anchorage, Alaska, USA

[3]North Slope Borough, Department of Wildlife Management, Utqiağvik, Alaska, USA

[4]Institute of Arctic Biology, University of Alaska Fairbanks, Alaska, USA

[5]Marine Mammal Pathology Services, Olney, Maryland, USA

[6]Alaska Department of Environmental Conservation, Anchorage, Alaska, USA

[7]U.S. Department of Agriculture, National Veterinary Services Laboratories, Ames, Iowa, USA

[8]Southeastern Cooperative Wildlife Disease Study, University of Georgia, Athens, Georgia, USA

*Corresponding author: 4210 University Drive, Anchorage, AK, 99508; 907-786-7174; aramey@usgs.gov

Any use of trade, firm, or product names is for descriptive purposes only and does not imply endorsement by the U.S. government.

Supplemental Figure S1. Maximum likelihood phylogeny depicting the estimated relationships among genetic sequences for N1 gene segment sequences for a H5 clade 2.3.4.4b highly pathogenic avian influenza viruses recovered from a polar bear (*Ursus maritimus*) inhabiting Alaska during 2023 (red font) and other closely related viruses from wildlife, poultry, and humans. Tip labels include standard abbreviations for states within the United States of America and Canadian provinces. Bootstrap support values ≥0.700 are presented. The scale bar represents the number of substitutions per site.

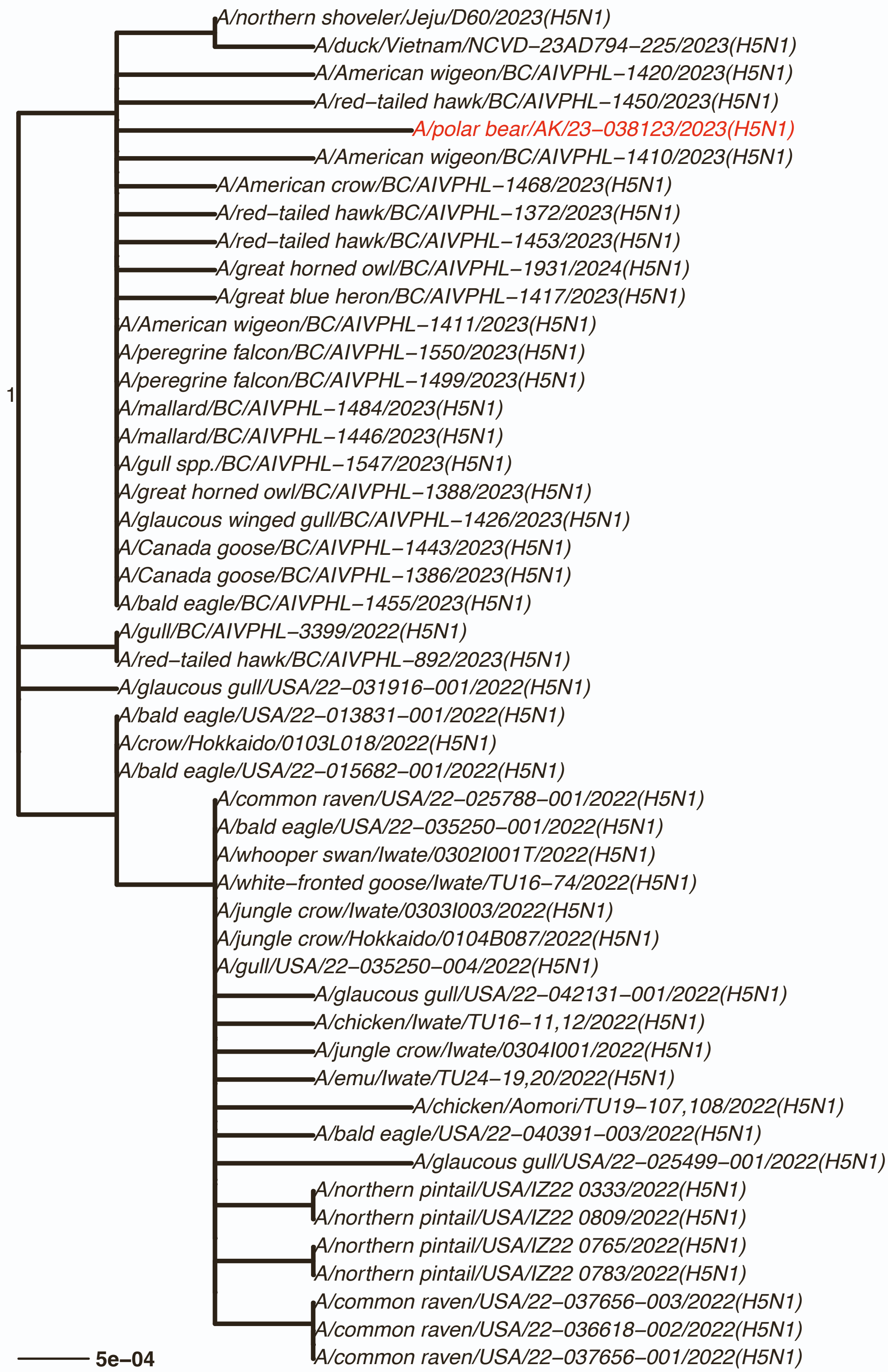

Supplemental Figure S2. Maximum likelihood phylogeny depicting the estimated relationships among genetic sequences for N5 gene segment sequences for a H5 clade 2.3.4.4b highly pathogenic avian influenza viruses recovered from a polar bear inhabiting Alaska during 2025 (red font) and other closely related viruses from wildlife, poultry, and humans. Tip labels include standard abbreviations for states within the United States of America and Canadian provinces. Bootstrap support values ≥0.700 are presented. The scale bar represents the number of substitutions per site.

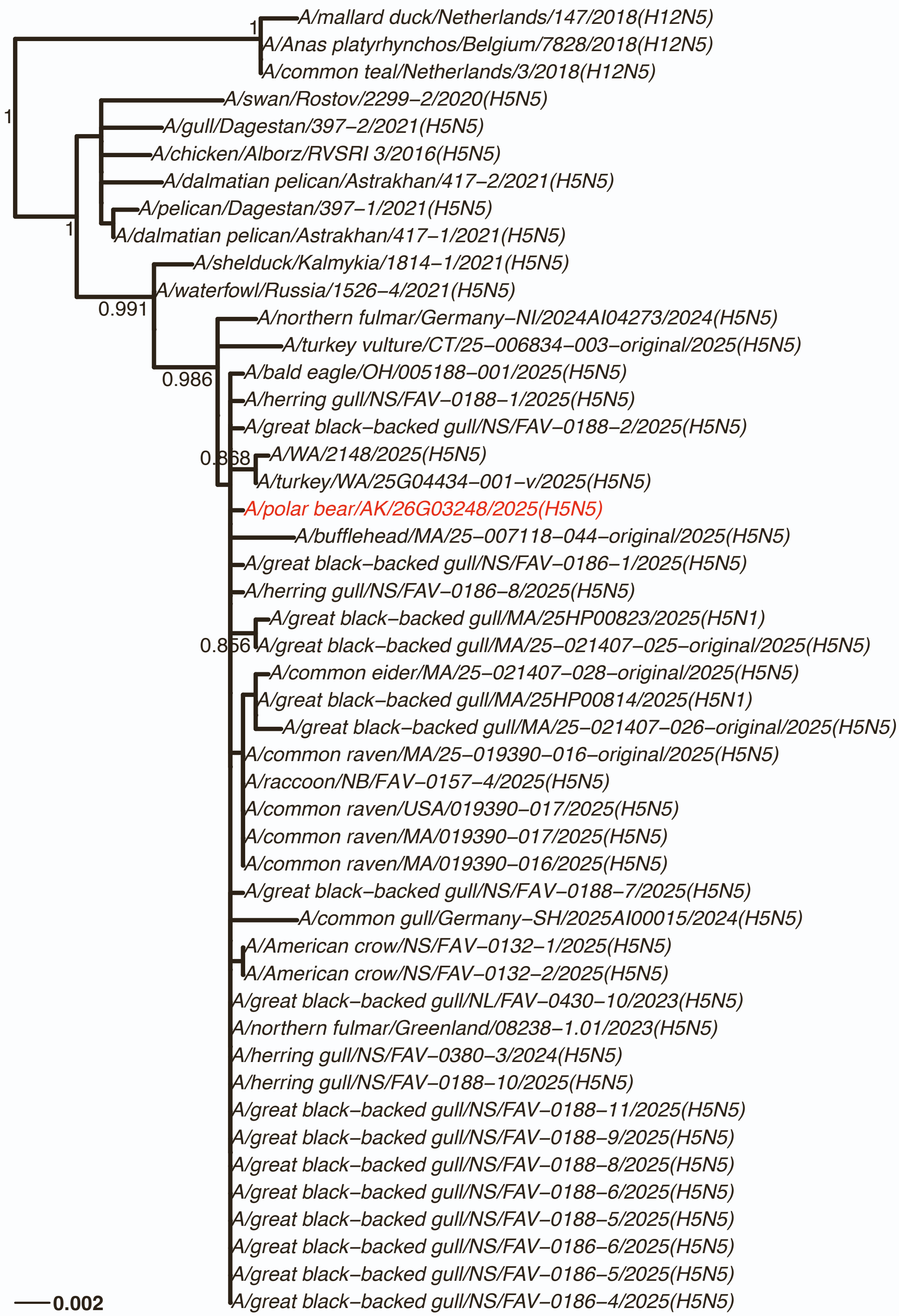

Supplement: Supplementary file 1 [file mmc1.pdf]
